# Polymer Optical Waveguide Sensor Based on Fe-Amino-Triazole Complex Molecular Switches

**DOI:** 10.3390/polym13020195

**Published:** 2021-01-07

**Authors:** Muhammad Shaukat Khan, Hunain Farooq, Christopher Wittmund, Stephen Klimke, Roland Lachmayer, Franz Renz, Bernhard Roth

**Affiliations:** 1 Hannover Centre for Optical Technologies, Leibniz University Hannover, 30167 Hannover, Germany; hunain.farooq@stud.uni-hannover.de (H.F.); bernhard.roth@hot.uni-hannover.de (B.R.); 2 Institute for Inorganic Chemistry, Leibniz University Hannover, 30167 Hannover, Germany; cwittmund@web.de (C.W.); stephen.klimke@acd.uni-hannover.de (S.K.); franz.renz@acd.uni-hannover.de (F.R.); 3 Institute for Product Development, Leibniz University Hannover, 30823 Hannover, Germany; lachmayer@ipeg.uni-hannover.de; 4 Custer of Excellence PhoenixD, Leibniz University Hannover, 30167 Hannover, Germany

**Keywords:** polymer optical sensor, microfabrication, temperature sensor, memory effect, iron-triazole complexes, hot embossing, maskless lithography

## Abstract

We report on a polymer-waveguide-based temperature sensing system relying on switchable molecular complexes. The polymer waveguide cladding is fabricated using a maskless lithographic optical system and replicated onto polymer material (i.e., PMMA) using a hot embossing device. An iron-amino-triazole molecular complex material (i.e., [Fe(Htrz)_2.85_(NH_2_-trz)_0.15_](ClO_4_)_2_) is used to sense changes in ambient temperature. For this purpose, the core of the waveguide is filled with a mixture of core material (NOA68), and the molecular complex using doctor blading and UV curing is applied for solidification. The absorption spectrum of the molecular complex in the UV/VIS light range features two prominent absorption bands in the low-spin state. As temperature approaches room temperature, a spin-crossover transition occurs, and the molecular complex changes its color (i.e. spectral properties) from violet-pink to white. The measurement of the optical power transmitted through the waveguide as a function of temperature exhibits a memory effect with a hysteresis width of approx. 12 °C and sensitivity of 0.08 mW/°C. This enables optical rather than electronic temperature detection in environments where electromagnetic interference might influence the measurements.

## 1. Introduction

Optical waveguide sensors measuring temperature are of keen interest for numerous applications in which electronic sensors are not applicable, for example, due to electromagnetic interference. Concepts based on optical waveguide principles rely on different sensing concepts, from the thermal expansion of the waveguide core material to Bragg gratings or the detection of bending losses [1]. Another approach is based on the insertion of molecular complexes as sensing material inside the core of optical waveguides. For example, molecular complexes featuring a spin transition from a low spin to a high spin state are attractive species [2,3,4]. These complexes can be used as molecular switches that change the spin state by external stimuli (i.e., changing ambient temperature, pH value, or exposure to light). Among the most promising representatives are iron-triazole complexes that produce spin transitions near room temperature [5,6]. During these transitions, various material properties can change (e.g., electrical, magnetic, or optical properties), which can then be utilized for sensing applications [7,8,9]. Over the years, multiple complex compounds have been investigated. For example, Letard et al. examined [Fe(NH_2_trz)_3_](Br)_2_⋅3H_2_O (with NH_2_trz = 4-amino-1,2,4-triazole) nanoparticles with a spin transition near room temperature, where a nanoparticle-size-dependent memory effect was reported [10]. Similarly, electronic devices with a memory effect based on the [Fe(Htrz)_2_(trz)](BF_4_) complex (with Htrz as 1,2,4-triazole) have been investigated [11,12,13]. The use of iron-based molecular complexes with spin-crossover properties for the measurement of pressure and temperature, as well as colorimetric sensors for the vapor phase detection of alcohols and toxic gasses, have been reported in [14,15,16,17]. By using the molecular complexes and combining them with polymer-optical waveguides, multiple sensing sensors can be realized (temperature, pressure, chemical concentration, etc.).

With regard to the fabrication of polymer-optical waveguide structures suited to realizing the aforementioned sensor devices, multiple lithographic techniques have been explored so far [18,19,20]. The laser direct writing (LDW) of structures on polymers using photoresist and two-photon polymerization have been demonstrated [21]. Additionally, the fabrication of three-dimensional polymer waveguide coupler structures on glass substrate using LDW for optical chips has been reported [22]. Similarly, the use of photoresist-coated wafer stamps containing waveguide structures replicated onto polymer material using hot embossing, employed for application in integrated photonics, has been reported [23,24,25]. Finally, multilayer waveguide structures fabricated using maskless lithography and hot embossing, laminated using optical adhesives, have been realized for optical sensing [26,27]. Among these methods, maskless lithography and hot embossing has proven to be both efficient and cost effective in manufacturing the required polymer structures [28,29].

In this work, we report an optical-waveguide-based sensor relying on [Fe(Htrz)_2.85_(NH_2_-trz)_0.15_](ClO_4_)_2_ (with 4-(amino)-1,2,4-triazole). The molecular complex is integrated in the waveguide core together with the core material. For this purpose, the waveguide core is fabricated from polymer material using maskless optical lithography. The cladding is realized by replication onto polymer using an in-house hot embossing system and then filled with the core material containing the molecular complex. As proof of concept, ambient temperature measurement is demonstrated to verify the basic functionality of the approach. Future work will focus on enhancement of the sensitivity and the detection of additional parameters such as strain, pH level, or humidity.

## 2. Fabrication of the Polymer Waveguide Cladding

The fabrication of PMMA waveguides is performed in a two-step process. First, a rib structure is created in photoresist coated on a silicon wafer substrate. Then, a lab-made hot embossing system is utilized to replicate the structures onto a PMMA sheet. For fabrication of the rib structure onto the silicon wafer, the wafer is coated with a resist onto the wafer surface using a standard spin-coating machine (WS-650SX223 6NPP-LITE, Laurell). The resist used is the hybrid co-polymer material Ormocomp (from Micro resist technology GmbH). The thickness of the resist layer is controlled by the spinning speed of the wafer. The silicon wafer used is 4 inches in diameter, with a thickness of 400 µm. Because the Ormocomp resist is highly viscous, to achieve a desired layer thickness, the sample must be coated at a high spinning speed. To facilitate this process, a thinner (i.e., Ormothin) is added to the Ormocomp resist to decrease the viscosity. To fabricate a waveguide with the desired groove depth of 20 µm, a ratio of 1:5 Ormocomp and Ormothin is used at a spinning speed of 4000 rpm of the spin coater. This results in the required layer thickness of approx. 20 µm, measured using a confocal microscope (Keyence VHX-7000) by determining the difference between coated and uncoated wafer surface areas. After coating, the wafer is prebaked at 80 °C for 2 min to crosslink the resist with the wafer surface.

**Figure 1:**
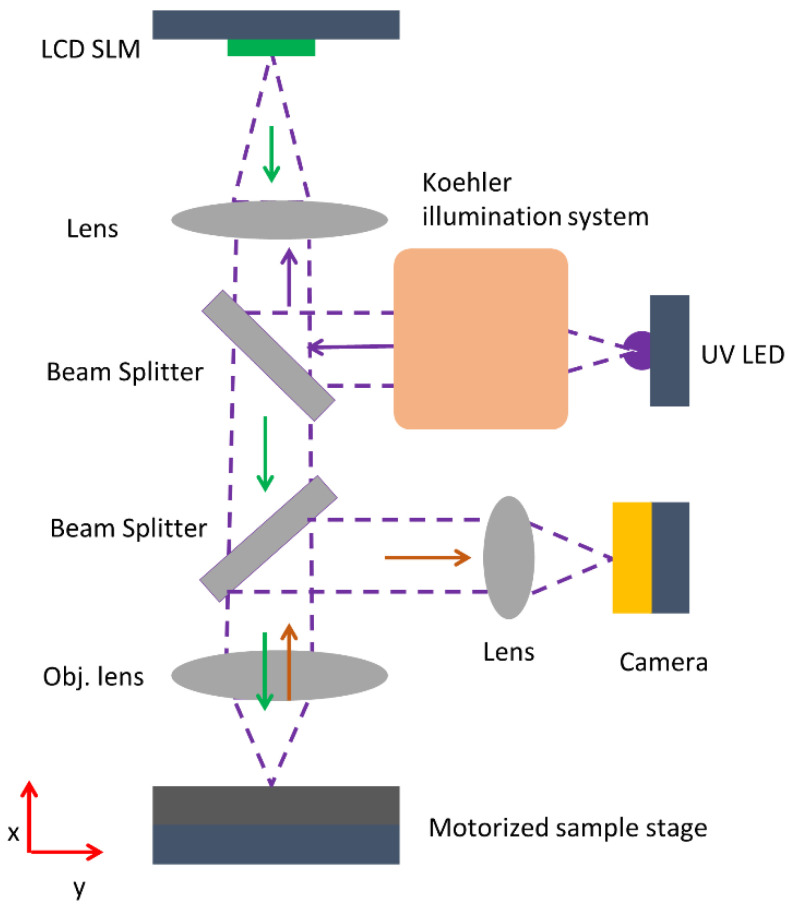
Scheme of the in-house maskless optical lithography setup used to fabricate the waveguide structures.

The wafer is then used in a maskless lithography system to fabricate grooves that serve as waveguide cladding in the subsequent step (Figure 1). The system relies on a reflective liquid crystal spatial light modulator (LCD SLM, Holoeye 6001) for pattern projection. A UV LED with a wavelength of 405 nm is used to illuminate the effective area of the SLM which features 1920 × 1080 pixels, with each pixel having a pitch of 8 µm. These pixels can be addressed electrically by displaying an image onto the SLM screen to create a certain pattern. The UV light is projected onto the SLM through a Koehler illumination system to homogeneously illuminate its effective area. The modulated light from the SLM screen is reflected and projected onto the resist-coated sample using a microscopic setup consisting of tube lenses and an objective lens. A CCD camera is installed in the setup to monitor the structures on the sample. The objective lens used is a plan-achromatic lens (from Zeiss GmbH) with 10× magnification and a numerical aperture (NA) of 0.31. The width of the fabricated waveguide grooves is controlled by the number of pixels in the pattern displayed on the SLM screen. We used 10 pixels of the SLM screen for the waveguide groove to achieve a width of approx. 8 µm on the resist (Figure 1). The UV light modulated in amplitude by the SLM is projected onto the sample for 30 s. The motorized sample stage is used to stitch multiple single exposure patterns on the resist in order to fabricate longer waveguides, as required here. A stitching error appears and is compensated by a predefined deflection in the position of the stage using a MATLAB program. The resulting stitching error observed is less than 1 μm, which is sufficiently small for the studies performed in this work. After exposure, the sample is baked at 115 °C to crosslink the cured resist with the wafer surface. It is then developed using OrmoDev for 90 s and subjected to a chemical bath with isopropanol. The depth and the width of the fabricated waveguide groove is determined using a confocal microscope.

**Figure 2:**
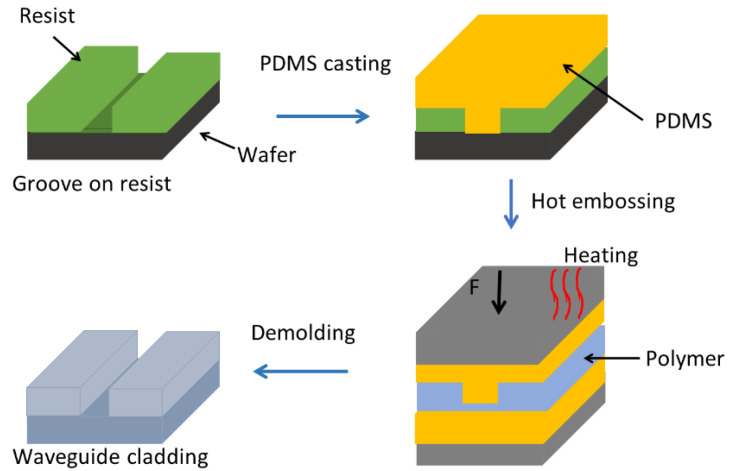
Schematic representation of the fabrication process of the waveguide cladding on polymer, showing intermediate steps.

As mentioned above, the fabricated waveguide stamp serves to replicate the groove structure onto PMMA in a lab-made hot embossing system, as can be seen in Figure 2. The latter consists of two heating plates with integrated heat-sensing elements and a manual pressure pump. The heating elements in the plates are connected to a control box containing an Arduino controller. Because the resist stamp is unstable at high temperatures and under mechanical stress, breakage of the stamp and defects in the waveguide structure occur during the embossing process. To overcome this, soft embossing is used [26]. For this purpose, a silicon elastomer (polydimethyl siloxane, PDMS) is employed to replicate the structure onto the PMMA. A silicon elastomer stamp is prepared from the resist stamp using PDMS casting. We used Elastosil 607 A/B with a ratio of 1:9 of the base and curing agent, respectively. For casting of PDMS, we prepared an aluminum casing to create a soft stamp with homogenous thickness. An inhomogeneous thickness will lead to an inhomogeneous pressure exerted onto the soft stamp and the polymer, resulting in deformed structures. The PDMS mixture is inserted into the casing and cured at room temperature for 24 h. The PDMS stamp is then removed from the resist stamp manually. Small tilts between the heating plates were compensated by an additional blank PDMS stamp placed on top of the polymer to distribute the applied force homogeneously over the polymer area (Figure 2). The stamps are then introduced into the hot embossing system so that the embossing process can be applied. The stamp and the polymer sheet are heated to an embossing temperature of approximately 140 °C, which is above the glass transition temperature of PMMA. After reaching the embossing temperature, the stamps are pressed against each other by manually applying the pressure using the pressure pump for a holding time of about 4 min. The PDMS stamp and PMMA foil are then cooled to a demolding temperature of 60 °C and manually demolded.

## 3. Waveguide Core and Molecular Complex

To detect a change in temperature, we used a molecular compound based on an iron-amino-triazole complex, that is, [Fe(Htrz)_2.85_(NH_2_-trz)_0.15_](ClO_4_)_2_. The complex undertakes a reversible spin transition and splitting of energy levels of d-orbitals through electrostatic interactions with the ligands. These transitions are affected by external factors such as change in temperature, among others. Synthesis of the complex was performed using the methodology described in Kröber et al. [6]. An amount of 0.395 g (5.7 mmol) of 1*H*-1,2,4-triazole and 0.026 g (0.3 mmol) of 4-(amino)-1,2,4-triazole are dissolved in 100 mL of ethanol. Similarly, 100 mL methanol and 0.735 g (2.0 mmol) of ferrous perchlorate hexahydrate and 0.013 g of ascorbic acid are dissolved. Both are mixed and stirred for 220 min. Afterward, a rotary evaporator is used to evaporate the solvents. An amount of 0.898 g (1.7 mmol) white powder material is obtained, corresponding to a yield of 73.3%, which upon cooling changes its color to purple.

**Figure 3:**
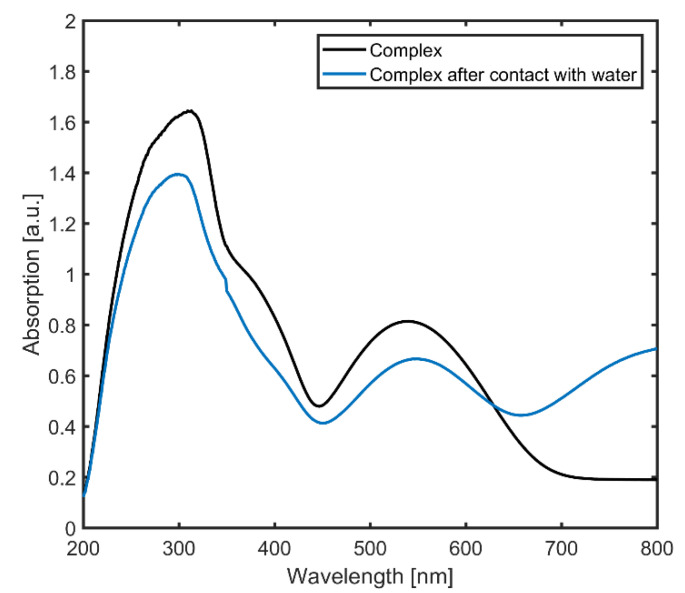
Solid-state UV/VIS spectrum of the [Fe(Htrz_)2.85_(NH_2_-trz)_0.15_](ClO_4_)_2_ complex.

The UV/VIS spectrum of the complex in the low-spin state exhibits two absorption bands located at around 314 nm and 513 nm (see Figure 3, black line). The first absorption band at 314 nm is due to the π–π* transition of the imine compound (R-C=N-R). The second absorption band at 513 nm is associated with the d–d transition. Adding water to the solid compound results in a spin transition from the low- to the high-spin state due to a shift of the hysteresis loop arising from changes in the interactions between two complex chains [30]. Additionally, a third absorption band can be found in the near-infrared range; however, this band lies outside the recording range of the measuring device, so the exact central wavelength could not be assigned (see Figure 3, blue line). The spin transition could also be triggered by heating the sample to slightly above room temperature, leading to similar UV/VIS spectra.

**Figure 4:**
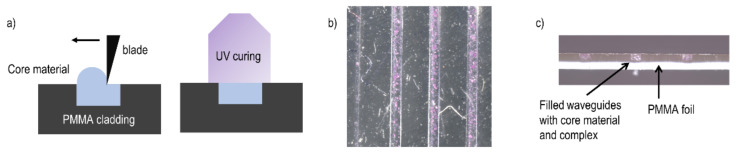
Fabrication scheme and image of created waveguide. (**a**) Waveguide core filling procedure, doctor blading, and UV curing; (**b**) Waveguide with molecular complex in the low-spin state (purple particles) embedded inside the core; (**c**) Side facet view of the fabricated waveguide structure. The images were obtained by confocal microscopy.

In the next step toward the realization of a sensor device, the molecular complex is mixed into the core material for the waveguide. This is required with regard to the detection of a change of the propagation of the light in the waveguide upon variation of the ambient temperature. For the waveguide core, a polymer material known as NOA68 from Nordland Inc., with a refractive index of 1.54, is used. The complex is added to the core material and the waveguide is filled by applying the mixture to the surface and doctor blading (see Figure 4a). The waveguide is then cured in a UV chamber with an illumination wavelength of 365 nm for 20 min until the NOA68 solidifies (Figure 4a,b). After curing, a perpendicular cut is applied using an automated blade cutter to smoothen the facets of the waveguide for light coupling (Figure 4c).

## 4. Results and Discussion

**Figure 5:**
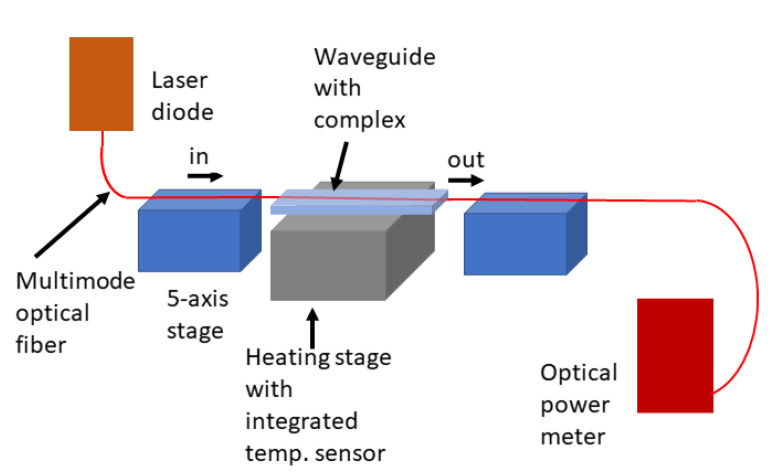
Optical setup used for measurement of the change in temperature versus temperature applied to the sample.

For the detection of temperature changes, the optical measurement setup shown in Figure 5 is used. The fabricated waveguide with the molecular complexes is placed on a heating stage with integrated heating cartridges and a Peltier element. The change in temperature of the stage is controlled by the power applied to the heating cartridges. The temperature is varied from 18 °C to 35 °C, with a step size of 0.01 °C, as this is the range of the expected spin-crossover transition. A multimode fiber-coupled laser diode at a wavelength of 638 nm is used to couple the light into the waveguide. This wavelength was selected for analysis because the absorption spectra of the complexes do not change too much near this wavelength upon varying the temperature or being in contact with water, as shown in Figure 3. For other wavelength regions (e.g., in the green or infrared region), the incident light may induce spin-crossover transitions, which is the case in most iron-triazole-based complexes, and will require detailed examination in future studies. A five-axis positioning stage is employed on both ends of the waveguide to align the optical fiber axis with the waveguide facet for in-coupling and out-coupling of the propagating light. The output fiber is connected to an optical power meter to measure the output power.

**Figure 6:**
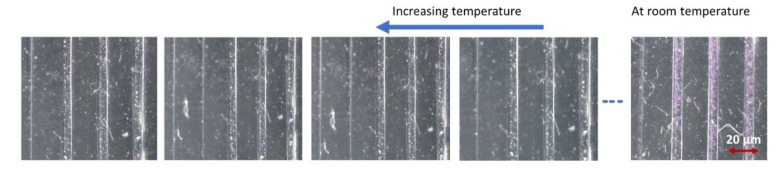
Image of the waveguide with amino-triazole complexes showing switching in color of the complex from pink-violet to white as the temperature increases from room temperature to approximately 35 °C.

As the temperature of the waveguide (and thus the complexes) increases, the color of the waveguide core changes from violet to white and reverses to pink-violet as the temperature cools down again (Figure 6). This occurs due to the spin crossover of the iron-triazole complexes and the accompanied change in the absorption bands in the visible range. This describes the process of the transitioning from a low-spin configuration to a high-spin configuration, or vice versa, without changing the spatial arrangement of the ligands.

**Figure 7:**
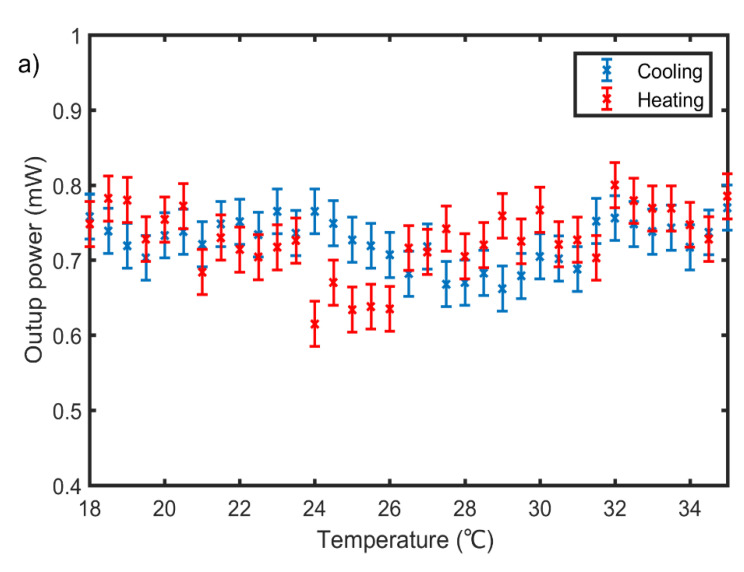

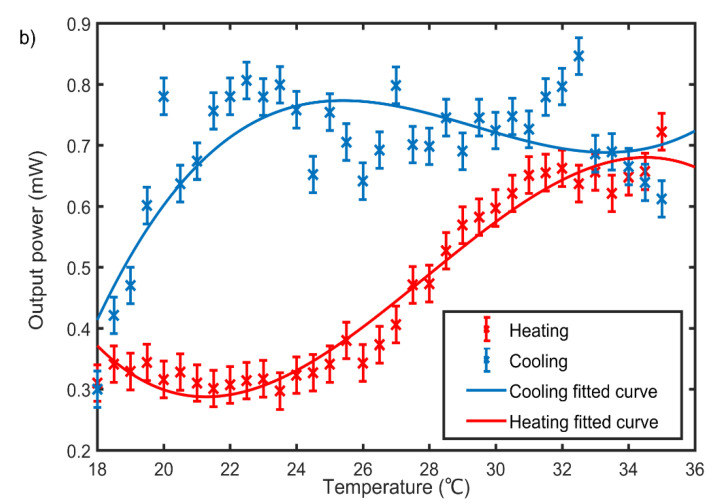
(**a**) Output power measured as a function of ambient temperature of the polymer waveguide without the molecular complex material. The residual variation of the power is neglected at this stage and will be studied in more detail in future studies. (**b**) Switching behavior of the polymer waveguide containing the iron complex as a function of ambient temperature, detected by measuring the output power during the heating and cooling cycles. The hysteresis memory effect is clearly visible.

The light propagating inside the waveguide experiences a change in the refractive index of the waveguide upon temperature change. This change in refractive index is visible in the output power measured on one end facet of the waveguide. When varying the temperature from 18 °C to 35 °C, forward current is applied to the cartridges in the heating stage and the heating cycle starts from room temperature to a temperature of 35 °C. After reaching 35 °C, the polarity of the current is changed to cool the element to a temperature of 18 °C. With the system used, temperatures up to 50 °C can be achieved, but the waveguide sample becomes unstable due to expansion of the PMMA waveguide structure. As such, a temperature range close to room temperature is used in the measurements. Additionally, a reference sensor is considered which consists of a polymer waveguide but without the Fe-amino-triazole complex. The transmitted optical power measured at the output facet of the reference sensor exhibits an almost constant behavior during heating and cooling cycles (Figure 7a). For the sensor with the molecular complexes, as the temperature increases from 18 °C to 35 °C, the output power measured at the power meter varies clearly from 0.385 mW to 0.775 mW. To deduce the sensitivity of the sensor, that is, the change in the output power upon a change in the temperature of the fabricated sensor, as defined in Equation (1), a quadratic curve fitting is applied in Figure 7b and the slope of the linear part of the curve is determined:

(1) $$\mathrm{sensitivity}=\frac{\left[∆P\right]\;}{\left[∆T\right]}.$$

Here, ∆P and ∆T denote the change in output power and temperature, respectively. We obtained a sensitivity = 0.08 mW/°C for the fabricated sensor element. A noticeable power change can be detected after minimal temperature variation of 0.5 °C, which is thus assumed to be the resolution of the sensor. The corresponding signal-to-noise ratio (SNR) is 18.24 dB and obtained from the average sensor signal S (Figure 7b) and the standard deviation of the reference signal N (Figure 7a), the latter being considered as noise, as defined in Equation (2):

(2) $$\mathrm{SNR}=\frac{\mathrm{Average}\;\mathrm{sensor}\;\mathrm{signal}\;S}{\mathrm{Standard}\;\mathrm{deviation}\;\mathrm{of}\;\mathrm{reference}\;\mathrm{signal}\;N}$$

Similarly, the output power measured at the output interface of the fiber shows a clear dependence on the temperature and a hysteresis memory effect, as can be seen in Figure 7. This response enables the complex to be used for the sensing of ambient temperature.

## 5. Conclusions

In this work, we presented a polymer-waveguide-based optical temperature sensor relying on [Fe(Htrz)_2.85_(NH_2_-trz)_0.15_](ClO_4_)_2_ embedded in a waveguide core. Fabrication of the structure is achieved by preparing a master tool using maskless lithography. A tool containing groove structures with a width and depth of 8 μm × 20 μm is created in photoresist. The structure is then replicated onto PMMA using a lab-made hot embossing system. A working tool is created by PDMS casting to produce a rib structure, which is finally replicated in PMMA. For filling of the waveguide core material into the grooves, NOA68 with embedded iron-triazole complexes is used. A spin crossover from low-spin state to a high-spin state, or vice versa, is possible with external stimuli—in our case, a change in temperature. The sample was heated and cooled with a step size of 0.01 °C in the range from 18 °C to 35 °C and the output power measured. As the temperature rises above room temperature, a color change of the complexes is observed, evidencing the spin transition. The output power recorded in the heating and cooling of the complex exhibits a hysteresis curve or a memory effect with a width of approximately 12 °C. The sensor exhibits a calculated sensitivity of 0.08 mW/°C with a temperature resolution of 0.5 °C and signal-to-noise ratio (SNR) of 18.24 dB. The concept enables optical temperature measurement without the influence of electromagnetic interference compared to electronic counterparts. The complexes featuring spin crossover can not only be used for novel sensor technology, but also for storage memory in the future. Versatile other applications can be expected, as laser-based technology which is generally well-established in basic and applied science may broadly be available at lower complexity and cost and combined with functional materials and modern additive manufacturing techniques for high throughput fabrication of integrated devices. In future studies, we will improve the achievable sensitivity of the temperature sensor through optimization of the amount of molecular complexes embedded in the waveguide core and the optimization of the fabrication technique and employ the scheme for measurement of different quantities stimulating spin-crossover transitions such as pressure, deformation, pH level, or humidity. Additionally, the influence of different spectral regions on inducing spin-crossover transitions will be studied systematically.

## Data Availability

The data presented in this study are available on request from the corresponding author.
